# *Lactobacillus rhamnosus* GG supernatant enhance neonatal resistance to systemic *Escherichia coli* K1 infection by accelerating development of intestinal defense

**DOI:** 10.1038/srep43305

**Published:** 2017-03-06

**Authors:** Xiaolong He, Qing Zeng, Santhosh Puthiyakunnon, Zhijie Zeng, Weijun Yang, Jiawen Qiu, Lei Du, Swapna Boddu, Tongwei Wu, Danxian Cai, Sheng-He Huang, Hong Cao

**Affiliations:** 1 Department of Microbiology, Guangdong Provincial Key Laboratory of Tropical Disease Research, School of Public Health, Southern Medical University, Guangzhou, 510515, China; 2 The First School of Clinical Medicine, Southern Medical University, Guangzhou, 510515, China; 3 Saban Research Institute, Children’s Hospital Los Angeles, University of Southern California, Los Angeles, 90027, USA

## Abstract

The objective of this study was to determine whether *Lactobacillus rhamnosus* GG culture supernatant (LCS) has a preventive effect against gut-derived systemic neonatal *Escherichia coli (E. coli*) K1 infection. The preventive effects were evaluated in human colonic carcinoma cell line Caco-2 and neonatal rat models. Our *in vitro* results showed that LCS could block adhesion, invasion and translocation of *E. coli* K1 to Caco-2 monolayer via up-regulating mucin production and maintaining intestinal integrity. *In vivo* experiments revealed that pre-treatment with LCS significantly decrease susceptibility of neonatal rats to oral *E. coli* K1 infection as reflected by reduced bacterial intestinal colonization, translocation, dissemination and systemic infections. Further, we found that LCS treated neonatal rats have higher intestinal expressions of Ki67, MUC2, ZO-1, IgA, mucin and lower barrier permeability than those in untreated rats. These results indicated that LCS could enhance neonatal resistance to systemic *E. coli* K1 infection via promoting maturation of neonatal intestinal defense. In conclusions, our findings suggested that LCS has a prophylactic effect against systemic *E. coli* K1 infection in neonates. Future studies aimed at identifying the specific active ingredients in LCS will be helpful in developing effective pharmacological strategies for preventing neonatal *E. coli* K1 infection.

In spite of great progress in anti-microbial therapy and supportive care, sepsis and meningitis remain a major cause of high mortality and severe neurological morbidity in neonates, especially in the preterm and very-low-birth-weight infants^1^,^2^,^3^. *Escherichia coli (E. coli*) K1 is the most predominant gram-negative bacteria that cause neonatal sepsis and meningitis^4^. The incidence of *E. coli* infections may further increase because of the recent emergence of antibiotic resistant *E. coli* strains. Furthermore, both clinical and experimental data suggest that the therapeutic efficacy of antimicrobial treatment alone is always limited for gram-negative bacillary meningitis^5^. A previous report demonstrated that prolonged neonatal administration of antibiotics is associated with increased risk of sepsis^6^. Therefore, it is necessary to develop alternate treatment strategies for preventing neonatal sepsis and meningitis.

Understanding and delineating the mechanism and the course of neonatal *E. coli* K1 sepsis and meningitis could provide a foundation for developing novel prophylactics. Although the exact mechanisms of *E. coli* K1–induced pathogenicity remain unclear, the natural course of *E. coli* K1 infection involving a series of steps as following have been established in detail: (a) gastrointestinal colonization by *E. coli* K1, often vertical transmission from the mother’s birth canal during delivery^7^,^8^; (b) *E. coli* K1 crosses the intestinal mucosal barrier and escape into the blood stream, then survive and multiply in the blood resulting in bacteraemia^9^; (c) finally, the bacteria transmigrate across the blood-brain barrier (BBB) and invade the central nervous system resulting in inflammatory responses and pathophysiological alterations such as pleocytosis and BBB injury that ultimately leads to neurological complications or death^10^. These steps indicate that the blockage of bacterial adherence to enterocyte and translocation across the intestinal barrier into the bloodstream would be a potential approach to prevent neonatal *E. coli* K1 sepsis and meningitis. Accumulating evidence shows that, probiotics exhibit protective effects on the intestinal mucosal barrier function, and are considered as an attractive option for preventing and/or treating *E. coli* K1 sepsis and meningitis. Further to this observation, our recent studies suggested that probiotics have a great potential to become a novel prophylactic for preventing neonatal bacteremia and meningitis^11^.

Probiotics are live bacteria which confer a beneficial effect on the host if administered in adequate amounts^12^. There is growing evidence that probiotics showed protective effect against a variety of disorders, such as obesity^13^, allergic asthma^14^, necrotizing enterocolitis^15^, diarrhea^16^, infection^17^,^18^ and cardiovascular diseases^19^. To date, many beneficiary effects of administering probiotics in gut associated diseases have been characterized, which includes maintenance of intestinal homeostasis, competitive exclusion of pathogens, promotion of mucin production, enhancement of intestinal barrier function, anti-inflammatory effects and immunomodulatory functions^20^,^21^. However, concern about the safety of live probiotics should be addressed, because of many up coming reports on increasing evidence of probiotic-associated infection in preterm infants and immunocompromised patients^22^,^23^,^24^. A randomised, double-blind, placebo-controlled trial demonstrated that in patients with predicted severe acute pancreatitis, probiotic prophylaxis with a multispecies probiotic preparation did not reduce the risk of infectious complications and was associated with an increased risk of mortality^25^. Furthermore, studies have revealed that *E. coli* Nissle 1917, a well known probiotic, exerts protective effect against mucosal disorders with considerable potential to induce gene mutations *in vitro* and cause DNA damage *in vivo*^26^,^27^. These adverse effects are associated with the development of colorectal carcinoma^27^. Additionally, a more serious problem was indicated by Million M *et al.,* who noticed that there are publication biases in probiotics related papers, because lots of smaller or deleterious results were not published, even when authors are directly sponsored by food industry^28^. Thus, it is mandatory to develop a safer alternative to the live probiotics for clinical applications.

More recently, probiotic-derived soluble factors (defined as “postbiotics”^29^) have been suggested to have beneficial properties as same as their original “parent”-live probiotics. Some active components have been identified from postbiotics, including short chain fatty acids, polyamines, polyphosphate, proteins and peptides. These active components have been implicated to exhibit a beneficial effect against several intestinal disorders through competition with pathogens, maintenance of intestinal barrier integrity and promoting immune function^30^,^31^,^32^,^33^. Administering postbiotics not only can avoid the potential risks associated with live microorganisms but also confers the same beneficial effects on the host. Thus, developing postbiotics as innovative health-promoting agents and their successful implementation in clinical medicine could revolutionize the modern drug therapeutics.

Based on the rationale mentioned above, we speculated that *Lactobacillus rhamnosus* GG culture supernatant (LCS) could have a protective effect against gut-derived *E. coli* K1–induced neonatal bacteremia and meningitis. To verify this speculation, human colonic carcinoma cell line Caco-2 and neonatal rats were pre-incubated with and without LCS and then exposed to *E. coli* K1. We found that pre-treatment with LCS could inhibit adhesion, invasion and translocation of *E. coli* K1 to Caco-2 monolayer as well as alleviate bacterial intestinal colonization, translocation, dissemination and systemic infection in neonatal rats. Furthermore, we observed that pre-incubation with LCS could promote the maturation of neonatal intestinal defense and thereby, enhance the resistance of neonatal rats to oral *E. coli* K1 infection. Overall, our data indicate that LCS has a potential to become an effective prophylaxis for neonatal sepsis and meningitis.

## Results

### Pre-treatment with LCS inhibited the adhesion and invasion of *E. coli* K1 to Caco-2

**Figure 1:**
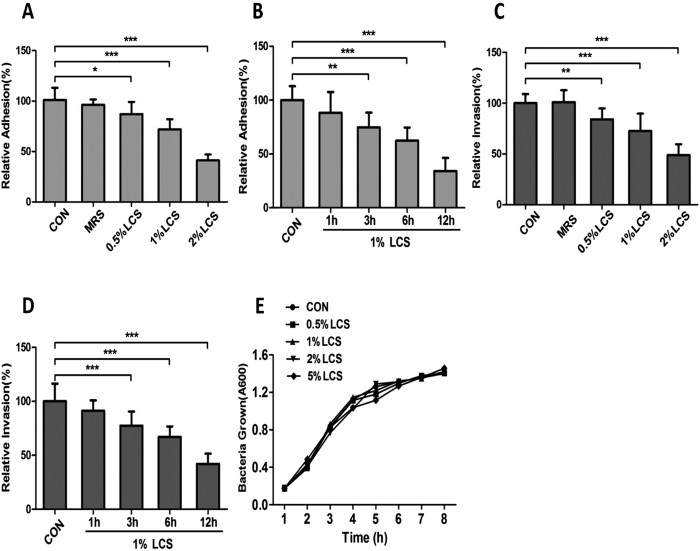
Pre-treatment with LCS significantly reduces adhesion and invasion of *E. coli* K1 to Caco-2. Caco-2 monolayers were incubated with cell culture medium, MRS or different concentrations of LCS for 3 h, or with 1% LCS for different time duration before infection. MRS (LGG’s medium) and cell culture medium were used as controls. Then *E. coli* K1 (MOI = 100) was added and incubated for 3 h. The numbers of associated bacteria (**A**,**B**) and intracellular bacteria (**C**,**D**) were determined. The results were expressed as a percentage of the control. Error bars indicate standard deviations. (**E**) Effect of LCS on the growth of *E. coli* K1 in BHI broth at different concentrations of LCS. Bacterial growth was monitored by measuring the absorbance of liquid cultures at 600 nm. *P < 0.05, **P < 0.01, ***P < 0.001.

Because adherence and invasion to intestinal epithelium are the pivotal steps for intestinal bacterial translocation and to enter the circulation resulting in a systemic infection, we firstly determined whether LCS has the inhibitory effect on adhesion and invasion of *E. coli* K1. Caco-2 monolayers were pre-incubated with different concentrations of LCS for 3 hours (h) before the bacterial infection. Monolayers treated with cell culture medium or MRS (LGG culture medium, not used, 2% in the cell culture medium without antibiotics) alone served as controls. Numbers of cell-associated bacteria and intracellular bacteria were determined. The data reveal that pre-incubation with LCS could dose- and time-dependently inhibits adhesion and invasion of *E. coli* K1 (Fig. 1A–D). Interestingly, we could not find any obvious inhibitory effects on adhesion and invasion of *E. coli* K1 on adding LCS 1 h after *E. coli* K1 infection (Data not shown). To examine whether LCS has antibacterial activity, we assessed the influence of LCS on the growth of bacteria cultured *in vitro* with brain heart infusion (BHI) broth. As shown in Fig. 1E, the growth curves of bacteria grown in BHI with or without LCS were similar. This result demonstrates that LCS has no lethal effect on *in vitro* growth of *E. coli* K1. Furthermore, the trypan blue stain assay showed that LCS has no detectable cytotoxicity on Caco-2 (data not shown). Overall, these data suggested that LCS can effectively inhibit adhesion and invasion of *E. coli* K1 but has no impact on *in vitro* growth of *E. coli* K1.

### Mucin is required for LCS-mediated inhibitory effect on adhesion and invasion of *E. coli* K1

**Figure 2:**
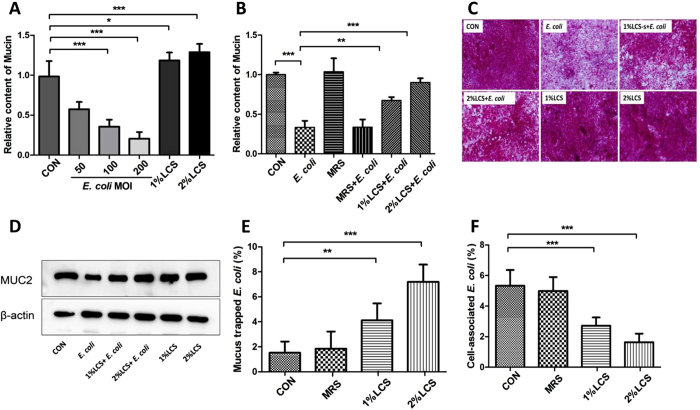
LCS inhibits adhesion and invasion of *E. coli* K1 via up-regulating mucin expression. Caco-2 cells were treated with various concentrations of *E. coli* K1 or LCS for 3 h, or pre-incubated with cell culture medium, MRS, or LCS for 3 h followed by *E. coli* K1 challenge. (**A**,**B**) The contents of mucin in cell lysates were determined by PAS assay as described in Methods. The results are expressed as relative content, taking the value for the control as 1. (**C**) Morphological alterations of mucin layers were determined by PAS staining. (**D**) Cells were treated as shown above for western blotting using an anti-MUC2 antibody. (**E**,**F**) Cells were pre-treated with various concentrations of LCS for 3 h and followed by *E. coli* K1 infection, cell-associated bacteria and mucus trapped bacteria were separated and counted as described in Methods. Data are expressed as the percentage of trapped *E. coli* K1 or adhered *E. coli* K1 among the added bacteria. *P < 0.05, **P < 0.01, ***P < 0.001.

Direct killing and competing adhesion sites with pathogen are two major mechanisms by which viable LGG inhibit bacterial adhesion and invasion. Thus, it is puzzling that how LCS exhibit an inhibitory effect on adhesion and invasion of *E. coli* K1 (Fig. 1A–D), without any steric-hindrance or direct killing effect on *E. coli* K1 (Fig. 1E). Mucin layer is an important barrier that separates the pathogen from enterocyte. We thus speculated that, mucin layer may play a pivotal role in LCS-mediated inhibitory effect on adhesion and invasion of *E. coli* K1. To test this hypothesis, we firstly evaluated the influence of LCS or *E. coli* K1 on production of mucin in Caco-2 monolayer using Periodic Acid Schiff (PAS) assay as described in Methods. As shown in Fig. 2A, infection with *E. coli* K1 markedly reduced the expression of mucin from multiplicity of infection (MOI) of 50 to 200 compared with the control. In contrast, LCS could significantly elevate mucin production in a concentration-dependent manner. We next examined whether LCS has any protective role against mucin-depletion effect of *E. coli* K1. Result shown in Fig. 2B suggested that pre-treatment with LCS could significantly alleviate *E. coli*-induced loss of mucin. Similar results were also observed with morphological alterations of mucin layers. As shown in Fig. 2C, untreated Caco-2 monolayer was covered with a purple, homogeneous and continuous mucin layer. After exposure to *E. coli* K1, however, the mucin layer became thinner and disrupted. In contrast, pre-incubation with LCS for 3 h could prevent from *E. coli* K1-induced disruption of the mucin layer. Furthermore, we evaluated the expression levels of MUC2 in each group using western blot analysis. As shown in Fig. 2D, pre-incubation with LCS alleviated *E. coli* K1-induced depletion of MUC2.

N-acetylcysteine (NAC) is an agent which could remove the mucin layer from intestinal mucosa^34^. Hence, we used NAC to explore whether the mucin layer is required for LCS-mediated inhibitory effect. Caco-2 monolayers were infected as described in the adhesion assay and then the bacteria that were trapped in mucin and adhering to Caco-2 monolayers were separated using NAC and counted as described in Methods. As shown in Fig. 2E and F, untreated Caco-2 mucin layer (CON) only trapped about 1.5 ± 0.9% *E. coli* K1, led to about 5.3 ± 1.0% *E. coli* K1 adhered to monolayer. However, when pre-treated with 2% LCS for 3 h before the *E. coli* K1 challenge, the mucin layer trapped about 7.2 ± 1.4% *E. coli* K1 and only 1.6 ± 0.56% *E. coli* K1 were detected on Caco-2 cell surface. These data indicated that LCS could induce mucin production to trap *E. coli* K1 and block bacteria from getting access to the intestinal epithelia.

### Pre-treatment with LCS abrogate the deleterious effects of *E. coli* K1 on intestinal integrity

**Figure 3:**
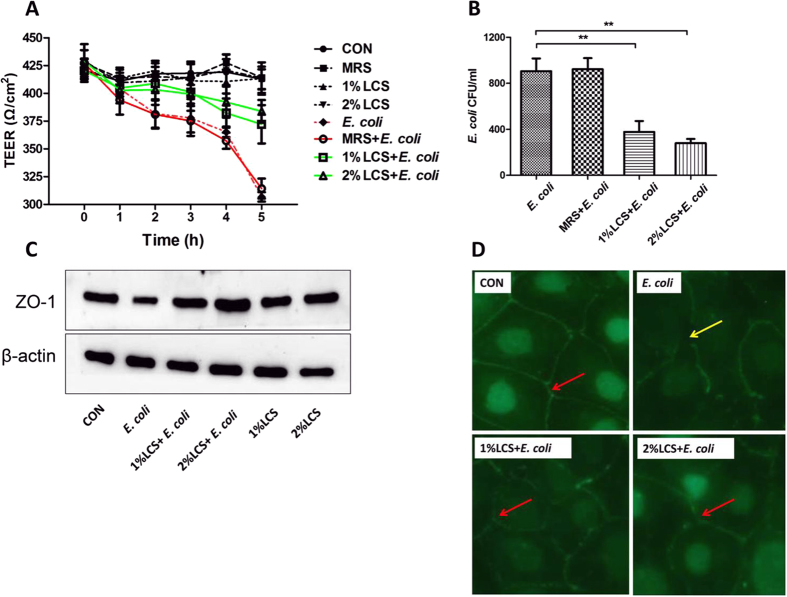
Pre-treatment with LCS attenuates *E. coli* K1-induced decreases in TEER and inhibit *E. coli* K1 translocation across Caco-2 monolayers. (**A**) TEER value of Caco-2 monolayers incubated with cell culture medium (CON), 2% MRS, 1% LCS and 2% LCS for 0–5 h without *E. coli* K1 infection or pre-treatment with LCS for 3 h prior to infection with *E. coli* K1 (MOI = 100) for 5 h. (**B**) The numbers of recovered *E. coli* K1 that were translocated to the basolateral medium after 5 h of apical infection. Results are presented as CFU/ml cell culture medium, **P < 0.01. Cells were treated as shown in Fig. 2C, (**C**) representative western blot for ZO-1 in Caco-2 lysates. β-actin bands were used as an indicator of protein loading. (**D**) Caco-2 monolayers stained for the tight junction protein ZO-1 (green, red arrows) was detected by a fluorescence microscope. Yellow arrows show the broken lining of ZO-1 expressions.

Once adhere to enterocyte, pathogen such as *E. coli* K1 could compromise intestinal integrity and translocation across the intestinal barrier into the blood stream, leading to systemic infection. Many studies have reported the protective effects of LCS against gut barrier injury caused by chemicals, such as alcohol, dextran sodium sulfate and hydrogen peroxide^35^,^36^,^37^,^38^,^39^. However, little is known about the roles of LCS on pathogen-induced intestinal barrier injury. Here, we examined whether LCS could abrogate the deleterious effects of *E. coli* K1 on intestinal integrity. Firstly, we explored whether treatment with MRS or LCS alone could reduce the trans-epithelial electrical resistance (TEER) values of Caco-2 monolayers. As shown in Fig. 3A, stable TEER values were observed in the control, MRS, 1% LCS and 2% LCS treated Caco-2 monolayers, suggesting that these factors have no detrimental role on intestinal integrity. However, infection with *E. coli* K1 could reduce the TEER values of Caco-2 monolayer in a time-dependent manner. In contrast, when pre-incubated with LCS for 3 h before infection, the reduction of TEER was alleviated (Fig. 3A). In parallel, the numbers of *E. coli* K1 translocated from the upper chamber to the lower chamber of transwell insert were dramatically reduced in LCS treated groups (Fig. 3B). These results suggest that LCS could reduce *E. coli*-caused gut barrier injury and prevent from intestinal translocation of *E. coli* K1.

To further investigate the protective effect of LCS on intestinal barrier function, we examined the zonula occludens-1 (ZO-1) expression in Caco-2 during *E. coli* K1 infection using western blot and immunofluorescent staining. As shown in Fig. 3C, *E. coli* K1 challenge resulted in a marked reduction of ZO-1 expression, but this detrimental role was abrogated by pre-incubation with LCS. Similar results were observed in immunofluorescent staining, which were shown in Fig. 3D. Non-infected Caco-2 exhibited continuous expression of ZO-1 between adjacent cells, and showed a typical “chicken-wire” shape with a continuous lining (red arrow). However, infection with *E. coli* K1 induced different degrees of morphological damage (yellow arrow). Pre-incubation with LCS significantly inhibited *E. coli*-induced destruction of ZO-1 tight junction morphology. Thus, we concluded that pre-treatment with LCS protected the intestinal barrier integrity against *E. coli* K1-caused injury.

### Effect of LCS on bacterial intestinal colonization, gut barrier injury and systemic infection during *E. coli* K1 infection in neonatal rats

**Figure 4:**
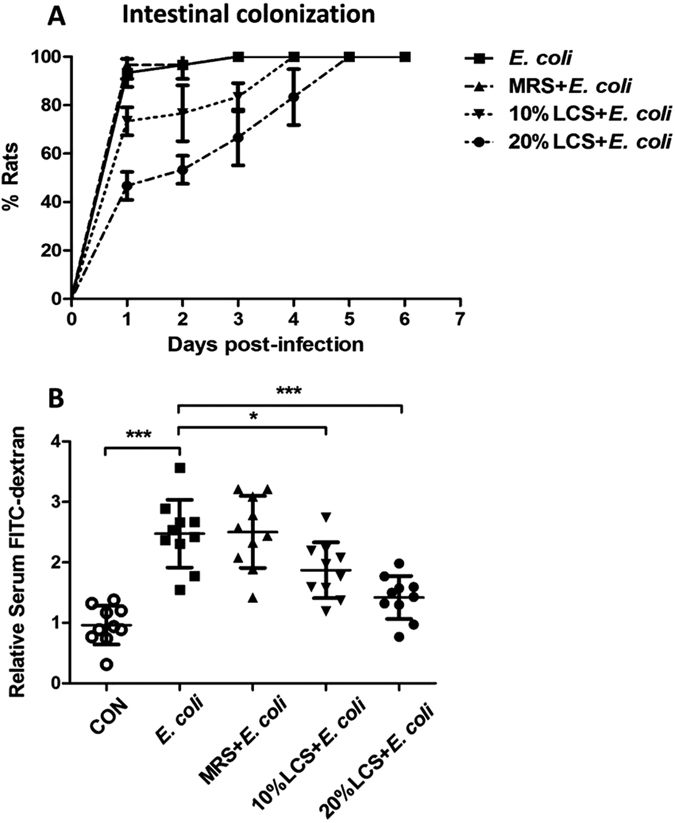
Pre-treatment with LCS delays the bacterial colonization and alleviate the *E. coli* K1-induced intestinal barrier injury. Rat pups were pretreated with PBS, MRS, 10% or 20% LCS for 3 days. Then all pups were infected with *E. coli* K1 (5 × 10^9^ CFU) by oral gavage. MRS (LGG’s medium) was used as a control. (**A**) The percentage of rat pups colonized with *E. coli*. All values represent the means of triplicate determinations. (**B**) Pups were treated as described above, intestinal permeability was evaluated using FITC-dextran. The results are expressed as relative content, taking the value for the control (un-infected pups) as 1. *P < 0.05, ***P < 0.001.

To examine the protective effect of LCS against neonatal *E. coli* K1 bacteremia and meningitis *in vivo*, we induced systemic *E. coli* K1 infection in neonatal rats via feeding live *E. coli* K1 as described previously^11^,^40^. In this animal model, the course of *E. coli* K1 infection mimics the natural route of intestinal colonization, translocation, bacteremia, sepsis and meningitis found in the human neonates. At the beginning of the experiments, all rat pups (1-day-postpartum, P1) from four litters were randomly divided into four groups and received PBS, 20% MRS, 10% or 20% LCS by oral gavage (twice a day for three days). Then systemic infection was induced by oral gavage with 5 × 10^9^ colony-forming unit (CFU) of *E. coli* K1 and the protective effects of LCS against bacterial intestinal colonization, gut barrier injury and systemic infection were evaluated as described in Methods. As shown in Fig. 4A, feeding *E. coli* K1 to PBS- or MRS-treated pups led to almost 100% intestinal colonization within 24 h after infection and persisted throughout the period of observation (6 days). In contrast, only 73% pups in 10% LCS treated group and 47% pups in 20% LCS treated group were colonized with *E. coli* within 24 h after infection. These results indicated that pre-treatment with LCS delayed the intestinal colonization of *E. coli* K1. Next, we evaluated the intestinal barrier permeability using fluorescein isothiocyanate (FITC)-dextran. Results showed that the pups which were pre-treated with LCS have lower serum levels of FITC-dextran than that in untreated pups (Fig. 4B), indicating that LCS could prevent *E. coli*-induced intestinal barrier injury.

**Figure 5:**
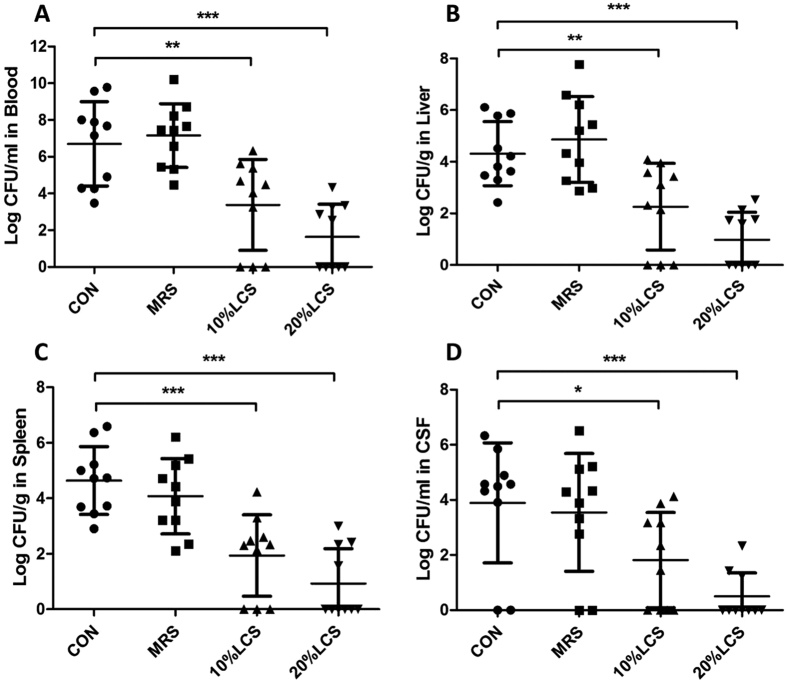
Inhibition of bacterial dissemination and systemic infection in neonatal rats pre-treated with LCS. Rats were treated as described in Fig. 4. *E. coli* K1 were recovered 60 hours after infection from the blood (**A**), liver (**B**), spleen (**C**) and CSF (**D**). All data were expressed as means ± standard deviation. *P < 0.05, **P < 0.01, ***P < 0.001.

The numbers of *E. coli* K1 in blood, liver, spleen and cerebrospinal fluid (CSF) were detected as described in Methods. Our results showed that *E. coli* K1 CFU counts in blood, liver, spleen and CSF were significantly decreased in rat pups that were pre-treated with LCS (Fig. 5A–D). Taken together, these data suggested that LCS could raise the resistance of neonatal rats to gut-derived *E. coli* K1 infection.

### LCS enhances the neonatal resistance to *E. coli* K1 infection via promoting postnatal maturation of intestinal defense

**Figure 6:**
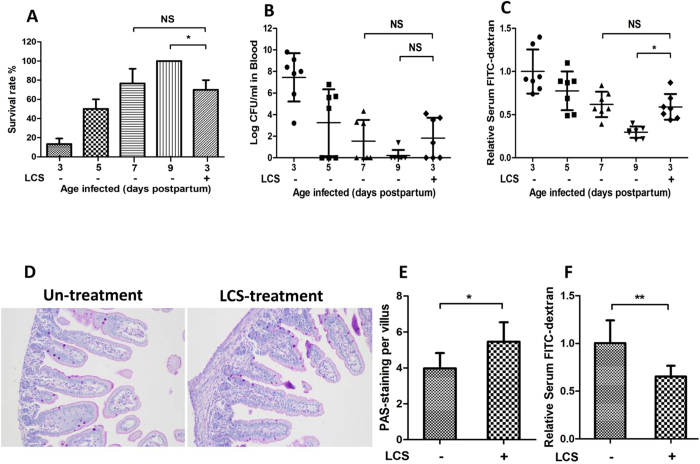
The resistance of LCS-treated P3 pups to *E. coli* K1 infection was similar to P7 pups. P3, P5, P7, P9 and LCS-treated (pre-treatment for 3 days) P3 pups were fed with 5 × 10^9^ CFU *E. coli* K1. (**A**) Percentage of survival (%) of pups. All the pups were monitored for a period of 7 days. (**B**) The number of *E. coli* K1 recovery from blood of neonatal rats. (**C**) Serum FITC-dextran was quantified as a measure of intestinal permeability. The results are expressed as relative content, taking the value for the P3 pups as 1. P1 pups were treated with or without 20% LCS for 3 days. (**D**) Ileum tissues were processed with PAS stain. (**E**) The percentage of PAS-positive cells per villus was calculated. (**F**) FITC-dextran assay was performed to evaluate the gut barrier formation *in vivo*. All data were expressed as means ± standard deviation. *P < 0.05, **P < 0.01.

**Figure 7:**
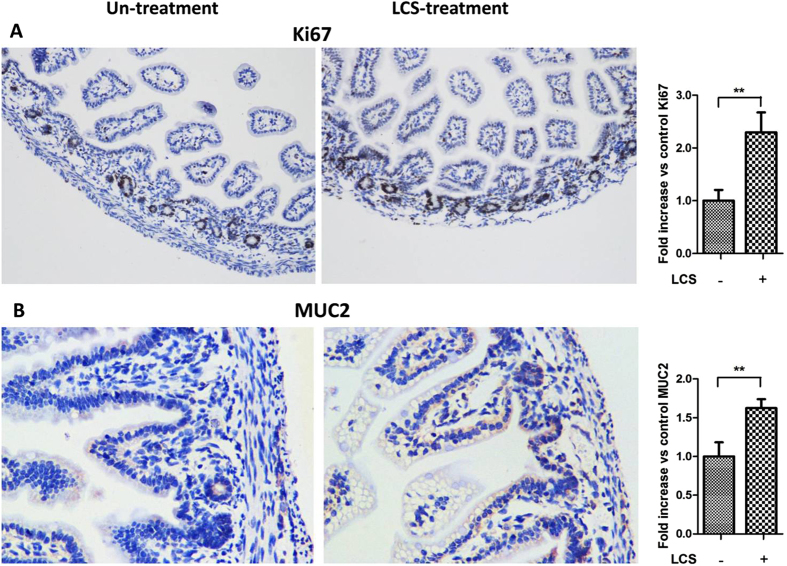
Pre-treatment with LCS enhances intestinal proliferation and differentiation of neonatal rats. P1 rat pups were treated with or without 20% LCS for 3 days. Immunostaining of Ki67 (**A**) and MUC2 (**B**) revealed an increase in the positive signals in intestine tissues of LCS-treated pups, compared with the untreated pups. Semiquantitative determination of Ki67 and MUC2 positive signals (right panel) was performed using Image J. **P < 0.01.

Birchenough GM *et al*. found that 9-day-old (P9) Wistar rat pups were more resistant to gut-derived *E. coli* K1 infection than two-day-old (P2) pups^40^. In our study, similar result was observed in Sprague Dawley (SD) rat pups. As shown in Fig. 6A–C, P3 pups, which were infected with *E. coli* K1 showed higher mortality, bacteremia and gut barrier permeability compared with P5, P7 and P9 pups. P9 pups were refractory to *E. coli* K1 infection with lowest mortality, bacteremia and intestinal barrier permeability. These results suggested that neonatal intestinal defense progress rapidly after birth. Importantly, we could notice that, on pre-treatment with LCS for three days, P3 pups could exhibit resistance to *E. coli* K1 infection similar with the P7 pups (Fig. 6A–C). Thus, we speculated that LCS has considerable potential to promote the maturation of neonatal intestinal defense. Based on these observations, we hypothesize that improvement in the maturation of intestinal defense could represent the underlying mechanism to explain the contribution of LCS to neonatal resistance against oral *E. coli* K1 infection. Further to test this hypothesis, we firstly explored whether administration with LCS promotes intestinal epithelial cell proliferation and differentiation via detecting the expression of Ki67 and MUC2 in the small intestine and colon respectively. As shown in Fig. 7A and B, the expression levels of Ki67 and MUC2 were significantly increased in the intestine tissues of LCS treated pups.

**Figure 8:**
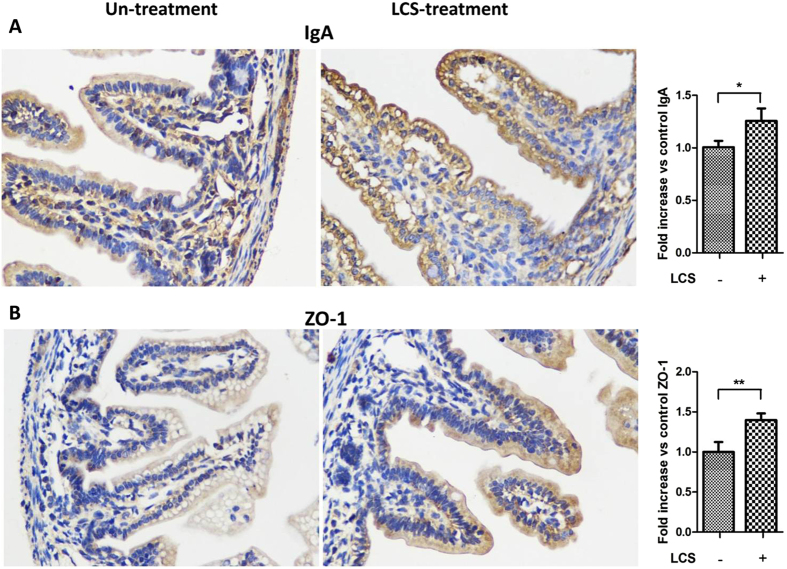
Pre-treatment with LCS promotes IgA production and ZO-1 expression in intestinal tissue of neonatal rats. P1 pups were treated as described in Fig. 7. Immunostaining of IgA (**A**) and ZO-1 (**B**) revealed an increase in the positive signals in intestine tissues of LCS-treated pups, compared with the untreated pups. Semiquantitative determination of IgA and ZO-1 positive signals (right panel) was performed using Image J. *P < 0.05, **P < 0.01.

Mucin, immunoglobulin A (IgA) and barrier function are key components of the intestinal defense. Thus, we next compared the production of mucin and IgA and formation of intestinal barrier function between LCS treated and untreated pups. Results showed that LCS treated pups have a higher expression level of mucin and IgA levels (Figs 6D,E and 8A). To evaluate the formation of intestinal barrier function, the gut permeability and expression of tight junction protein ZO-1 were detected. Intestinal permeability assay showed that LCS treated pups have lower intestinal permeability than that in untreated pups (Fig. 6F). Meanwhile, immunohistochemical staining showed that pre-treatment with LCS increased ZO-1 expression on the membrane of ileum (Fig. 8B). Overall, these results suggested that LCS could accelerate the development of neonatal intestinal defense.

## Discussion

In the present study, we intended to make a strategy shift from treatment to prevention of the neonatal systemic *E. coli* K1 infection through the use of probiotic culture product to enhance the intestinal defense, which is a unique preventive barrier against natural *E. coli* K1 infection. Our data showed that pre-treatment with LCS is able to delay *E. coli* K1 intestinal colonization, inhibit bacterial translocation and dissemination, and reduce bacteremia and meningitis in neonatal rats. These results suggested that administration with LCS enhances the resistance of neonatal rats to *E. coli* K1 infection. Further study found that LCS promotes intestinal proliferation and differentiation, accelerate intestinal barrier formation and increase mucin and IgA production in the gut of neonatal rat. These data indicated that neonatal administration with LCS accelerates the maturation of intestinal defense and confers a high resistance to intestinal infection. To the best of our knowledge, this is the first study reporting such beneficial effect of probiotic culture product on maturation of neonatal intestinal defense. Collectively, our data not only indicated that LCS has a great potential in preventing neonatal *E. coli* K1 sepsis and meningitis, but also has a potential to become an effective prophylaxis for immature intestine-associated diseases, such as inflammatory bowel disease, infectious enteritis and necrotizing enterocolitis.

Probiotics secrete many kinds of antimicrobial substances that inhibit pathogenic infection by direct killing effect, including bacteriocins, reuterin, organic acid, hydrogen peroxide and some heat-resistant small peptides^41^. It has been established that the antimicrobial activity of LGG is executed entirely through secreted cell-free supernatants^41^. In this study, we have found the inhibitory effect of LCS on bacterial adhesion and invasion. This effect, however, is unlikely to execute via direct inhibition on the growth of bacteria, because the concentrations of LCS used here, did not show any extracellular antimicrobial activity (Fig. 1E). In contrast, the anti-adhesion mechanism was based on indirect enhancement of mucin production that separated the pathogen from Caco-2 cells (Fig. 2E and F). Because LCS has no direct antibacterial activity to pathogens, it is unlikely to develop the microbial resistance. Meanwhile, this indirect mechanism makes LCS exert a preventive effect on most of gut-derived pathogenic infections rather than only *E. coli*. Furthermore, we found that LCS could also promote mucin production in the intestinal tract of neonatal rats (Fig. 6D and E), which is much significant during the neonatal period. Firstly, during neaonatal period, a protective intestinal mucus barrier always not fully developed^40^, and the up-regulated mucin production will form a physical barrier to protect the underlying epithelium from the attachment of pathogens. Secondly, mucin could provide carbon, nitrogen, and sulfur source for intestinal microbiota, especially *Akkermansia muciniphila*, which could through a turnover of mucus (degrade mucins and simulate mucin production), maintain the mucus thickness to protect the intestinal barrier^42^.

Intestinal epithelial barrier is formed and maintained by tight junction complexes, which plays a significant role as access point that checks bacterial translocation across the cell barrier. Disruption of the integrity of this barrier occurs in several diseases, such as inflammatory bowel disease^43^, necrotizing enterocolitis^44^ and certain bacterial and virus infections^45^. Numerous studies have reported the protective effect of live probiotics on the pathogen-induced tight junction injury *in vitro* and *in vivo*. For example, Johnson-Henry KC *et al*. showed that live LGG, but not heat-inactivated LGG protected polarized MDCK-I and T84 epithelial cell against Enterohemorrhagic *E. coli*-caused changes in TEER, dextran permeability, and redistribution of ZO-1 and claudin-1^46^. However, evidence on probiotic-derived product exerting protective effect against pathogen-caused tight junction injury is still lacking. In the present study, we observed that pre-treatment with LCS could alleviate *E. coli* K1-induced reduction of TEER values and ZO-1 expression in Caco-2 monolayer (Fig. 3A,C and D). These results indicated that LCS could prevent from *E. coli* K1-induced tight junction damage, as well as convincingly suggested that LGG culture supernatant has the similar protective effect on intestinal epithelial barrier as live LGG.

The immature intestinal defense in the neonatal period may provide opportunities for pathogenic translocation across the gut barrier into the blood stream, leading to systemic inflammation and infection^15^,^44^,^47^. In our study, we observed that feeding of *E. coli* K1 to P3 pups led to about 87% death within 7 days, with the higher intestinal permeability and more serious bacteremia than that in P5, P7 and P9 pups (Fig. 6A–C). The same phenomenon occurs in humans, because human neonates are also most susceptible to *E. coli* K1 infection during the early neonatal period^48^. Importantly, these results indicated that the intestinal defense is undergoing rapid maturation after birth. Interestingly, when pre-treated with LCS for three days, P3 pups exhibited the same resistance to oral *E. coli* K1 infection as P7 pups (Fig. 6A–C), indicating that LCS has potential to promote maturation of neonatal intestinal defense. Subsequently, we found that expression levels of Ki67 and MUC2, the maker of cell proliferation and intestinal differentiation respectively^49^, were significantly higher in LCS treated pups than those in untreated pups (Fig. 7A and B). Moreover, we observed that LCS could modulate important intestinal defense including ZO-1, mucin, IgA and barrier permeability (Figs 6D–F, 8A and B). These data suggested that LCS is able to accelerate the maturation of neonatal intestinal defense, and thereby reduce the susceptibility of neonates to *E. coli* K1 infection. These results were consisted with a previous study that, neonatal mice which colonization with live LGG had more sophisticated intestinal functions and were less susceptible to dextran sodium sulfate-induced colitis^50^ than the mice without LGG colonization. In another study, Ravi M. Patel *et al*. reported that neonatal colonization of mice with live or heat-killed LGG accelerates maturation of intestinal barrier function by promoting claudin 3 expression^44^. However, the authors found that administration with high-dose live LGG (10^9^ CFU/day for 7 days) lead to an increased mortality in 1-week-old mice. This paper highlight the safety concerns about the application of live probiotics in neonates. Indeed, according to the regulations of Food and Drug Administration in the United States, routine use of live organisms to immunocompromised premature infants is prohibited^51^. In this study, we found that LGG culture supernatant has shown a progressive effect on maturation of neonatal intestinal functions similar to live LGG, and thus support the application of probiotic-derived factors to replace live probiotic to avoid the potential risks if necessary.

Gut microbiome is largely subjected to dynamic changes after birth, and temporally take part in maturation of the intestinal immune system^52^. A recent study done by Deshmukh HS *et al*. demonstrated that a balanced intestinal microbiota plays key roles in neonatal resistance to *E. coli* K1 sepsis^52^. Thus, it is interesting to explore the effect of LCS on formation of neonatal intestinal microbiota. We speculated that LCS has a great potential to promote development of neonatal intestinal microbiota. Two rational bases supported this hypothesis. Firstly, it is established that besides protecting the enterocyte from pathogenic assault, mucus also provide glycan-dependent anchoring sites and nutrients to intestinal commensal microbiota^53^. Thus, it is possible that LCS-induced mucin production may help the colonization of commensal bacteria. Secondly, IgA, the important defense in intestinal mucosa to prevent the pathogen from attaching to the enterocyte, has been demonstrated that could promote the establishment of microbiota composition and maintains the diversity of microbiota^54^. Moreover, research has shown that IgA is able to maintain the homeostasis of gut flora using an “immune exclusion” strategy to exclude the pathogenic microbes and control the commensal microbes^55^. Thus, induced IgA expression by LCS may play an important role in formation, development and maintenance of intestinal microbiota composition. Further study should focus on exploring the relationship between LCS and formation of neonatal intestinal microbiota.

In the present study, we suggested that LCS has a promising application in preventing neonatal intestinal infection. However, it is unclear which LCS components exert the beneficial effects. LCS is a complex mixture containing lots of substances, including lipids, organic acids, proteins and other small molecules. Due to the complexity and uncertainty, it is necessary to keep an eye on the side effects of LCS. To date, p40 and p75 are two most characterized proteins purified from LGG-derived soluble factors and both of them have beneficial effects on intestinal barrier functions. However, p40 showed more potent effects than p75^56^. More recently, it has been established that p40 can induce IgA and MUC2 production in intestinal tissues through active epidermal growth factor receptor (EGFR)^57^,^58^. Furthermore, using sodium dodecyl sulfate-polyacrylamide gel electrophoresis (SDS-PAGE) analysis, we also detected two bands, ~40 KDa and ~75 KDa, exist in LCS in our study (data not shown). In accordance with our findings, it is reasonable to speculate that p40 may be the major active ingredient in LCS to exhibit the beneficial effects towards infectious pathogens.

In summary, our data suggested that LCS could prevent neonatal gut-derived *E. coli* K1 infection through promoting the maturation of neonatal intestinal defense. We believed that LCS has a great potential to become an alternative treatment option for preventing neonatal *E. coli* K1 sepsis and meningitis. Furthermore, our data also confirmed that supernatant from certain probiotic could exert beneficial effects similar with their live probiotic counterparts. Further identification of the active ingredients in LCS is required to develop newer prophylaxis for preventing neonatal sepsis and meningitis.

## Methods

### Ethics statement

All research involving animals has been approved by the ethics committee and performed strictly according to the guidelines for animal care in Southern Medical University (SMU, Guangzhou, China). Timed-pregnant SD rats were obtained from Animal Experimental Center of SMU and bred in-house. All supplements including food, water, and enrichment were autoclaved, and animals were kept in the animal facility. All surgeries were performed under anesthesia with ketamine and lidocaine, and utmost efforts were taken to minimize suffering.

### Bacterial strains, cell lines and cultures

The probiotic strain LGG (ATCC 53103) and pathogen *E. coli* K1 were kindly provided by Prof. Sheng-He Huang (University of Southern California, USA). *E. coli* K1 is a clinical isolate [*E. coli* RS218 (O18:K1:H7)] with rifampicin-resistance from the CSF of a neonate with meningitis^11^. LGG was grown in De Man, Rogosa, and Sharpe (MRS) broth (Oxoid, Hampshire, UK) at 37 °C for 24 hours. *E. coli* K1 was grown for 14 h at 37 °C in brain heart infusion (BHI) broth in the presence of rifampicin (100 μg/ml). LCS was prepared by centrifugation at 10,000 rpm for 1 minute (m) at 4 °C and dual filtration through 0.22 μm millipore filters. To avoid the detrimental effect of acidic compounds, LCS was concentrated using 3 kDa spin columns and diluted into cell culture medium or PBS at appropriate pH value (7–8) for subsequent experiments. To exclude the potential impact of probiotic culture medium, MRS were processed as LCS and served as controls in certain experiments. Caco-2 was purchased from Shanghai Institute of Cell Biology (Shanghai, China) and routinely cultured in Eagle’s Minimum Essential Medium with 10% heat inactivated fetal bovine serum and streptomycin (100 mg/ml) and penicillin G (50 mg/ml) at 37 °C in 5% CO_2_.

### Adhesion and invasion assays

Caco-2 cells were grown in a 24-well tissue culture plate (10^5^ cells per well) at a minimum of 12 days to allow them fully differentiate^11^. Before the adhesion assays, Caco-2 were pre-incubated with cell culture medium, 2% MRS, 0.5% LCS, 1% LCS or 2% LCS (dilution in cell culture medium) for 3 h. After treatment, cell monolayers were washed with phosphate buffered saline (PBS) three times and examined under the microscope. No morphologic changes were observed. Adhesion assays were performed in Caco-2 as previously described^11^,^59^. In brief, approximately 10^7^ CFU *E. coli* K1 were added to Caco-2 monolayer with a multiplicity of infection (MOI) = 100. The monolayers were incubated for 3 h at 37 °C. After incubation, the monolayers were washed three times with PBS and lysed by 0.5% Triton X-100 for 8 m. The bacteria were collected and enumerated by BHI agar plates with rifampicin (100 μg/ml). Each assay was performed in triplicate wells and repeated three times. The results were expressed as a percentage of the control (*E. coli* infection only).

For invasion assays, Caco-2 monolayers were infected with *E. coli* K1 as described as above. To eliminate any extracellular bacteria, the monolayers were incubated with experimental medium containing gentamicin (100 μg/ml) for 1 h at 37 °C. Then monolayers were washed three times with PBS and lysed by 0.5% Triton X-100 for 8 m. Intracellular bacteria were determined as mentioned above. Each assay was performed in triplicate wells and repeated three times. The results were expressed as a percentage of the control (*E. coli* infection only).

We used the NAC (Sigma-Aldrich, St. Louis, USA) to quantify *E. coli* K1 which was trapped in mucin or adhered to Caco-2 cells^34^. Caco-2 monolayers were infected with *E. coli* K1 as described in adhesion assays. Then infected monolayers were incubated with NAC (10 mM in PBS containing 0.2 μM CaCl_2_, 0.5 mM MgCl_2_ and 15 mM glucose) to remove the mucin layer (contains *E. coli* K1). Cells were washed four times with PBS to remove NAC and lysed with 0.5% Triton X-100 for 8 m. The number of associated bacteria was enumerated by BHI agar plate with rifampicin (100 μg/ml). To evaluate the bacteria trapped into mucus, removed mucin layers were collected and plated on BHI agar plates with rifampin (100 μg/ml) for counting. Each assay was performed in triplicate wells and repeated three times. Data are expressed as the percentage of trapped *E. coli* K1 or adhered *E. coli* K1 among the added bacteria.

### Bacterial translocation and TEER measurements

Caco-2 cells were grown in Transwell inserts (6.5 mm diameter, 3 μm pore size, Corning Costar Corp., USA) for at least 21 days to differentiate and to form tight junctions. This polarized monolayer mimics the pathogen translocation across the gut barrier (upper chamber) into the blood circulation (lower chamber). To determine whether LCS have protective effect on intestinal barrier against *E. coli* K1 infection, Caco-2 monolayers growth on Transwell insert were pre-incubated with cell culture medium, 2% MRS, 1% LCS, or 2% LCS for 3 h at 37 °C and 5% CO_2_. Then *E. coli* K1 (MOI = 100) were added to the upper chamber of Transwell inserts. TEER values were measured at 0, 1, 2, 3, 4, and 5 h post infection using a Millicell electrical resistance apparatus (EVOMAX, World Precision Instruments, USA). The bacteria translocated from the upper chamber to the lower chamber at 5 h post infection were quantified by plating on BHI agar with rifampicin (100 μg/ml) and incubated at 37 °C overnight.

### PAS assay

Caco-2 monolayers grown in 6-well plate were treated with different levels of *E. coli* K1 (MOI = 50, 100 or 200) or LCS (1% or 2%) or infected with *E. coli* K1 as mentioned in the adhesion assay. Then treated monolayers were collected and lysed in lysis buffer to obtain soluble ingredients. Mucin production in soluble fractions was measured as described by Wang LH *et al*.^58^. In brief, 0.1% periodic acid was added to each sample and incubated for 2 h at 37 °C. Then Schiff reagent was added and incubated for 30 m in dark at room temperature. Optical density of each sample was assessed using a microtitre plate reader set at 550 nm. All samples were analyzed in triplicate.

### PAS staining

Caco-2 cells grown in 24-well plate were infected with *E. coli* K1 as described in the adhesion assay. Then monolayers were harvested and fixed in 4% paraformaldehyde at room temperature for 20 m. PAS staining was performed according to the manufacturer’s instructions of PAS kit (Solarbio Science & Technology Co., Ltd, Beijing, China). The PAS-stained wells were counterstained with hematoxylin and observed using light microscopy. For PAS staining of intestinal tissues, 0035 μm sections were fixed in 4% formaldehyde and paraffin-embedded. PAS staining was conducted as described above.

### Neonatal rat model of hematogenous *E. coli* K1 systemic infection

Systemic *E. coli* K1 infection was induced using specific pathogen free SD rats as described previously with minor modifications^11^,^40^. Litters were retained with their natural mothers after birth. Four litters (10 pups per litter) were pooled and randomly distributed into four groups. Pups were fed with 100 ul of PBS, 20% MRS, 10% LCS or 20% LCS (dilution in PBS) twice a day for three days. Then all pups were gavage with 100 μl of *E. coli* K1 (5 × 10^9^ CFU). Sixty hours post infection, blood, liver and spleen were extracted aseptically after anaesthetizing the rats with ketamine and lidocaine. CSF samples were collected as described previously^60^. Bacteria were quantified in homogenized tissues by serial dilution culture on BHI agar plates with rifampicin (100 μg/ml). Gastrointestinal tract colonization of *E. coli* K1 was determined at 24 h intervals by culture of perianal swabs on MacConkey’s agar as described previously^61^.

Intestinal permeability was determined as previously described^62^. Briefly, pups were given FITC dextran (MW 4000 at 60 mg/100 g, Sigma) by gavage 4 h before sacrifice. Blood was collected from heart puncture and FITC concentrations were measured using a fluorescence spectrophotometer at an excitation wavelength of 485 nm and emission wavelength of 535 nm.

### Histopathological examination and immunohistochemical staining

For the immunohistochemical staining, 5 μm paraffin-embedded sections were deparaffinized and antigen was retrieved. Sections were blocked in 1% normal goat serum and incubated overnight at 4 °C with antibody specific for MUC2, ZO-1, Ki67 and IgA (all from Abcam, Cambridge, UK) respectively. The primary antibodies were visualized using horseradish peroxidase (HRP)-coupled second antibodies with 50 mM Tris-HCl buffer (pH 7.4) containing DAB (3,3′-diaminobenzidine) and H_2_O_2_, and the sections were lightly counterstained with hemotoxylin.

Quantification of immunoreactive signal was performed using National Institutes of Health image analysis software Image J^63^. In brief, the red-green-blue (RGB) bitmap images were firstly converted to 8-bit grayscale, then the threshold was modulated to display only positive signals and eliminate the background. The same cutoff value of threshold was used to analyze all the slides stained concurrently. The staining intensity measurements were calculated for the total area (total intensity/mm^2^). A total of five sections for each group were analyzed. We expressed the results as the relative area, taking the value of the control (untreated P3 pups) as 1.

### Western blotting analysis

To assess the expression of ZO-1 and MUC2 in Caco-2, cells were collected and lysed on ice in radio-immunoprecipitation assay buffer and boiled at 100 °C for 10 min. Equal amounts of proteins were separated on SDS polyacrylamide gels and transferred onto polyvinylidene difluoride (PVDF) membranes (Millipore). The membranes were incubated with a rabbit anti-ZO-1 antibody (1:200) or rabbit anti-MUC2 antibody (1:2000). Expression of primary antibodies was visualized using HRP-coupled second antibodies and enhanced chemiluminescence reagent kit (Bio-Rad Laboratories, USA). A goat polyclonal anti-β-actin antibody (1:1500) was used as a loading control. Each western blot assay was repeated at least three times.

### Immunofluorescence analysis

Caco-2 monolayers were fixed in 4% paraformaldehyde for 10 min at room temperature. Cells were blocked in 5% normal goat serum in PBS for 1 h at room temperature. Then monolayers were probed with a rabbit anti-ZO-1 antibody for 12 h at 4 °C followed by incubation with Alexa Fluor 568-coupled goat anti-rabbit secondary antibody (Invitrogen, Carlsbad, CA) at room temperature in dark for 1 h. Slides were mounted and observed using fluorescence microscopy (Nikon Eclipse: TE 2000-E, Japan).

### Statistical Analysis

Data are shown in mean ± standard deviation and analyzed by one-way analysis of variance (ANOVA) tests. All statistical analyses were carried out using SPSS 13.0 (SPSS Inc., Chicago, IL, USA) and *P* < 0.05 was considered to be statistically significant.

## Additional Information

**How to cite this article**: He, X. *et al. Lactobacillus rhamnosus* GG supernatant enhance neonatal resistance to systemic *Escherichia coli* K1 infection by accelerating development of intestinal defense. *Sci. Rep.* **7**, 43305; doi: 10.1038/srep43305 (2017).

**Publisher's note:** Springer Nature remains neutral with regard to jurisdictional claims in published maps and institutional affiliations.
